# 

**Published:** 1826-11

**Authors:** 


### MONTHLY LIST OF MEDICAL BOOKS.

[*No books can be entered on this List except those sent to us for the purpose; as, in the list hitherto transmitted, the names of works have frequently been given as published, which have not appeared for weeks, or even months, after.*]

An Essay on Morbid Sensibility of the Stomach and Bowels, as the Proximate Cause or Characteristic Condition of Indigestion, Nervous Irritability, Mental Despondency, Hypochondriasis, &c. &c. To which are prefixed, Observations on the Diseases and Regimen of Invalids, on their Return from hot and unhealthy Climates. By James Johnson, M.D.—London, 1827.

Manual of Gymnastics.—Royal 18mo. 6d.

Observations on the Preparatory Education of Candidates for the Degree of Doctor of Medicine, in the Scottish Universities. Humbly submitted to the Consideration of His Majesty's Commissioners for Visiting the Universities and Colleges of Scotland.—1826.

On Galvanism, with Observations on its Chemical Properties and Medical Efficacy in Chronic Diseases, with Practical Illustrations: also Remarks on some auxiliary Remedies. With Plates. By M. La Beaume.—London, 1826.

A Small Tract, consisting of Practical Observations on the following Subjects:—1. On the present State of Medicine. 2. On the Causes which impede the Progress of Medicine. 3. On Pulmonary Consumption. 4. On the Treatment of Wounds and Ulcers, and Hemorrhage. 5. Observations on Fractures, Dislocations, &c. 6. On the London Pharmacopœia. By Richard Walker, Surgeon-Apothecary, Oxford.—1825.

Manual of Pathology; containing the Symptoms, Diagnosis, and Morbid Characters of Diseases: together with an Exposition of the different Methods of Examination, applicable to Affections of the Head, Chest, and Abdomen. By L. Martinet, D.M.P. Translated with Notes and Additions, by Jones Quain, A.B.—London, 1826.

### METEOROLOGICAL JOURNAL,

### NOTICES.

*The continuation of* Mr. Brodie's *Paper on the Testicle will be given in our next.*

*Various other Papers, which have been unavoidably postponed, are also in the press.*

*We should be much obliged to the Author of the Paper entitled* "Observations on the Interior Structure and Economy of the Conglobate Glands," *if he would allow us to prefix his name.*

